# Supplementary material for: A Polylactide-Based Micellar Adjuvant Improves the Intensity and Quality of Immune Response
Source: Pharmaceutics. 2022 Jan 3;14(1):107. doi: 10.3390/pharmaceutics14010107 (PMC8778782; doi:10.3390/pharmaceutics14010107)
Supplement: Supplementary file 1 [file pharmaceutics-14-00107-s001.zip › pharmaceutics-1489735-supplementary.pdf]

pharmaceutics

Supplementary materials

A Polylactide-Based Micellar Adjuvant Improves the Intensity and Quality of Immune Response

Myriam Lamrayah [1,*], Capucine Phelip [1], Céline Coiffier [1], Céline Lacroix [1], Thibaut Willemin [1], Thomas Trimaille [2,*] and Bernard Verrier [1]

[1] Laboratoire de Biologie Tissulaire et Ingénierie Thérapeutique (LBTI), Institut de Biologie et Chimie des Protéines (IBCP), CNRS UMR 5305, Université Lyon 1, Université de Lyon, 69367 Lyon, France; capucine-phelip@gmail.com (C.P.); celine.coiffier@ibcp.fr (C.C.); celine.fish@free.fr (C.L.); thibaut.willemin@gmail.com (T.W.); bernard.verrier@ibcp.fr (B.V.)
[2] Laboratoire Ingénierie des Matériaux Polymères (IMP), CNRS UMR 5223, Université Lyon 1, Université de Lyon, 69622 Villeurbanne, France
* Correspondence: myriam.lamrayah@ibcp.fr (M.L.); thomas.trimaille@univ-lyon1.fr (T.T.)

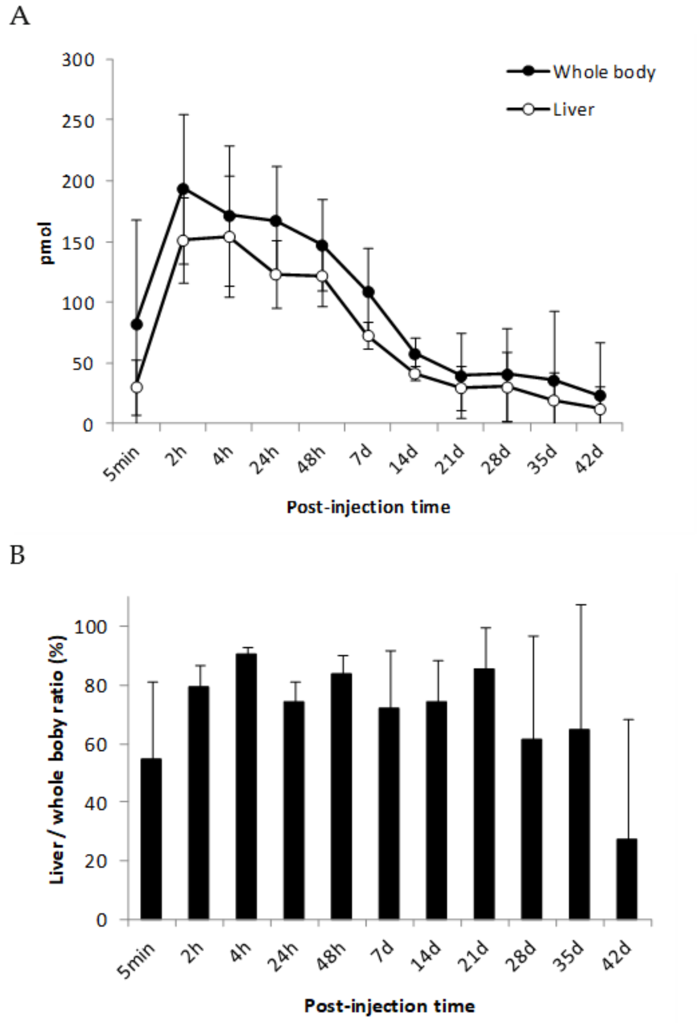

Figure S1. Distribution of DiR fluorescent micelles after their administration by intravenous route. (**A**) whole body and liver fluorescence vs. post-injection time; (**B**) liver to whole body fluorescence ratio vs. post-injection time.
